# Hypoxia-Activated Prodrug Derivatives of Carbonic Anhydrase Inhibitors in Benzenesulfonamide Series: Synthesis and Biological Evaluation

**DOI:** 10.3390/molecules25102347

**Published:** 2020-05-18

**Authors:** Emilie Anduran, Ashok Aspatwar, Nanda-Kumar Parvathaneni, Dennis Suylen, Silvia Bua, Alessio Nocentini, Seppo Parkkila, Claudiu T. Supuran, Ludwig Dubois, Philippe Lambin, Jean-Yves Winum

**Affiliations:** 1 Institut des Biomolécules Max Mousseron (IBMM) UMR 5247 CNRS, ENSCM, Université de Montpellier, 34296 Montpellier CEDEX 05, France; emilie.anduran@enscm.fr (E.A.); nandakumar683@gmail.com (N.-K.P.); 2 The M-Lab, Department of Precision Medicine, GROW–School for Oncology, Maastricht University, 6200 MD Maastricht, The Netherlands; ludwig.dubois@maastrichtuniversity.nl; 3 Faculty of Medicine and Health Technology and Fimlab Ltd., University of Tampere and Tampere University Hospital, 33520 Tampere, Finland; ashok.aspatwar@tuni.fi (A.A.); seppo.parkkila@tuni.fi (S.P.); 4 Department of Biochemistry, Cardiovascular Research Institute Maastricht (CARIM), Maastricht University Medical Centre, 6200 MD Maastricht, The Netherlands; d.suylen@maastrichtuniversity.nl; 5 Neurofarba Department, Section of Pharmaceutical Sciences, Università degli Studi di Firenze, 50019 Sesto Fiorentino (Florence), Italy; silvia.bua@unifi.it (S.B.); alessio.nocentini@unifi.it (A.N.); claudiu.supuran@unifi.it (C.T.S.)

**Keywords:** hypoxia-activated prodrug, carbonic anhydrase, inhibitors, sulfonamides, hypoxic tumour

## Abstract

Hypoxia, a common feature of solid tumours’ microenvironment, is associated with an aggressive phenotype and is known to cause resistance to anticancer chemo- and radiotherapies. Tumour-associated carbonic anhydrases isoform IX (hCA IX), which is upregulated under hypoxia in many malignancies participating to the microenvironment acidosis, represents a valuable target for drug strategy against advanced solid tumours. To overcome cancer cell resistance and improve the efficacy of therapeutics, the use of bio-reducible prodrugs also known as Hypoxia-activated prodrugs (HAPs), represents an interesting strategy to be applied to target hCA IX isozyme through the design of selective carbonic anhydrase IX inhibitors (CAIs). Here, we report the design, synthesis and biological evaluations including CA inhibition assays, toxicity assays on zebrafish and viability assays on human cell lines (HT29 and HCT116) of new HAP-CAIs, harboring different bio-reducible moieties in nitroaromatic series and a benzenesulfonamide warhead to target hCA IX. The CA inhibition assays of this compound series showed a slight selectivity against hCA IX versus the cytosolic off-target hCA II and hCA I isozymes. Toxicity and viability assays have highlighted that the compound bearing the 2-nitroimidazole moiety possesses the lowest toxicity (LC_50_ of 1400 µM) and shows interesting results on viability assays.

## 1. Introduction

Intratumoral heterogeneity, a main feature of solid tumours, is one of the causes of the intractability of cancers [1]. There are multiple mechanisms driving tumor heterogeneity, including genetic, epigenetic and microenvironmental factors such as hypoxia [2]. The presence of oxygen deprivation areas (typically less than 1%), defined as hypoxic domains, and resulting from inadequate tumor vascularization, have been identified in a wide variety of human tumours [3]. The adaptive cellular response to low oxygen tensions is coordinated by hypoxia inducible transcription factors HIF-1, which activate gene expression programs controlling multiple responses [4]. Among them are found the change in glucose metabolism towards anaerobic glycolysis (Warburg effect), which causes a decrease in the pH of the tumour microenvironment [5]. Several key proteins and buffer systems [6], including monocarboxylate transporters (MCTs), isoforms of anion exchanger, Na^+^/HCO_3_^–^ co-transporters, Na^+^/H^+^ exchangers, and carbonic anhydrases (CAs) isoforms IX and XII are involved in this pH regulatory process to maintain a physiological intracellular pH accompanied with extracellular acidification [7]. This acidosis strongly contributes to the malignant progression, aggressive phenotype, and resistance to therapy (chemotherapy and radiation) of the cancer cells, leading to a poor prognosis regardless of treatment [8].

The hypoxic microenvironment of solid tumors has attracted significant attention as a target which can be exploited in drug design for the development of novel anticancer or imaging agents [9].

Two approaches have been considered in the literature: the first approach consists of the inhibition of molecular targets necessary for the survival of hypoxic cells, particularly carbonic anhydrase IX and XII. A wealth of research depicts carbonic anhydrases isoform IX (hCA IX) and hCA XII as biomarkers and therapeutic targets for various cancer types, and both of these enzymes are associated with cancer progression, metastasis, and impaired therapeutic response. As examples, head and neck tumours as well as oral cancers, which are highly hypoxic, were reported to overexpress hCA IX [10,11,12,13]. The development of small molecules as specific CA IX inhibitors represents a successful field with several potent inhibitors reported so far [14,15,16,17]. The second approach is based on the exploitation of the redox potential, between hypoxic and normoxic areas for the development of prodrugs that activate selectively in a highly reducing hypoxic environment (Hypoxia Activatable Prodrugs, HAP) [18]. HAPs are hypothesized to improve the therapeutic index of drugs that are ineffective against tumor cells in hypoxic microenvironments. The potential of HAPs is often evaluated clinically in combination with other cancer treatment (chemotherapy or radiotherapy) to affect both the normoxic and hypoxic fraction of the tumour [18,19,20].

Over the last two decades, many HAPs have been documented [18]. The molecular motifs used as cleavable entities are all susceptible to bioreduction, mainly by enzymatic processes (e.g., reductases) by a mono- or di-electronic process depending on the enzyme involved. The most frequently found in the literature are *N*-oxide, quinone, or nitroaromatic derivatives, which have been the subject of in-depth studies up to the clinical stage [18,19,20]. Nitroaromatic HAPs have been described for targets such as poly ADP ribose polymerase (PARP) inhibitors [21], protein kinase RNA–like endoplasmic reticulum kinase (PERK) inhibitors [22], tyrosine kinase (TK) inhibitors [23] and also for different types of nanosystems [24,25]. Few numbers of carbonic anhydrase IX inhibitors (CAIs) have been designed using this HAP approach [26,27,28,29,30]. As part of an effort to discover novel small-molecule inhibitors of hCA to enhance cancer therapy, we report herein a new class of 2- and 5-nitroimidazole, nitrofuran, nitrothiophene and nitrogen mustards- (alkylating agents) based bio-reducible drugs harboring a benzenesulfonamide to target hCA IX.

## 2. Results

### 2.1. Chemistry

**Scheme 1:**
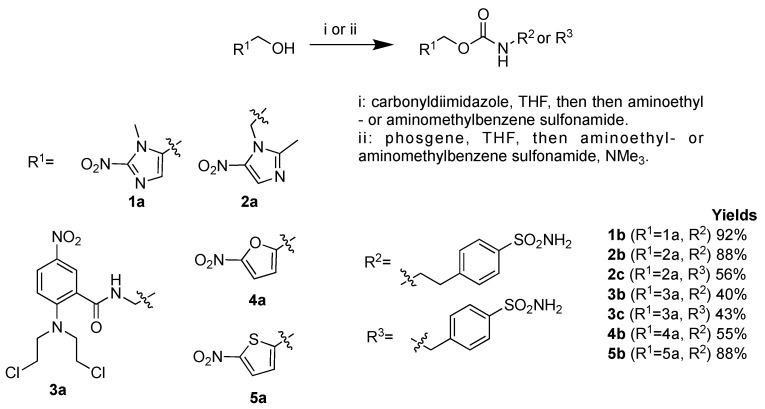
Synthesis of hypoxia-activated prodrug carbonic anhydrase IX inhibitors (HAP-CAIs) **1b**–**4b** and **2c**–**3c**.

2- and 5-nitroimidazole, nitrofuran, nitrothiophene and nitrogen mustard were conjugated with benzene sulfonamides using a carbamate linker. Because of the low reactivity observed, the introduction of the carbamate linker proved to be challenging for some derivatives and two approaches were used to achieve the synthesis of these inhibitors starting from the nitroaromatic alcohols: (i) a reaction with carbonyldiimidazole to access to the carbamoyl imidazole derivatives that reacted, in a one-pot reaction, with aminomethyl- or aminoethyl- benzenesulfonamide, or (ii) a reaction with phosgene to yield to the chloroformates which were then reacted with aminomethyl- or aminoethyl- benzenesulfonamide in the presence of triethylamine (Scheme 1) [31]. Compounds **1b**–**5b** and **2c**–**3c** were isolated with yields ranging from 43% to 88% and characterized extensively by spectroscopic and spectrometric methods (see materials and methods part).

### 2.2. Carbonic Anhydrase Inhibition Assay

The hypoxia-activated prodrug carbonic anhydrase IX inhibitors (HAP-CAIs) reported here were assayed using the CO_2_ hydrase assay against three physiologically relevant human CA isoforms, the cytosolic hCA I and II and the transmembrane, tumor-associated hCA IX (Table 1) [32]. 

The clinically used sulfonamide acetazolamide (**AAZ**, 5-acetamido-1, 3, 4- thiadiazole-2-sulfonamide) has been taken along as standard in these measurements. All compounds acted as inhibitors against the three isoforms hCA I, II and IX, although with variable potency (Table 1). Against the abundant, cytosolic isoform hCA I compound **3c** showed weak inhibition potency (2180 nM). Compound **1b** showed moderate inhibition activity towards hCA I (166.7 nM) and hCA II (30.6 nM) isoforms and higher inhibition (7.6 nM) towards hCA IX, while compounds **2b** and **2c** strongly inhibited all CA isoforms (K_I_ ranging from 2.3 to 14.1 nM) with no differences observed in the selectivity ratios hCA II over hCA IX (**2b** = 0.35, **2c** = 0.28). Compounds **3b** and **3c** showed a moderate inhibition of hCA IX, whilst compound **3b** binding to hCA II was very tight (3.1 nM). The large difference in the binding capacity or selectivity ratios (**3b** = 0.09, **3c** = 0.94) of compounds from the same family (nitrogen mustard) supports the substitution effect on the carbamate linker. Compounds **4b** and **5b** showed moderate inhibition activity towards hCA I, respectively (84 nM) and (64.7 nM), while they strongly inhibited hCA II and hCA IX (K_I_ ranging from 5.7 to 19.8 nM). Nevertheless, considering the difficulty of obtaining small compounds with a better affinity for the tumor-associated isozyme (hCA IX) over hCA II, the selectivity obtained for these series is comparable or better than that of the clinically used CA inhibitor acetazolamide **AAZ**.

### 2.3. Stability of Carbamate Linker under Acidic Conditions

The stability to chemical hydrolysis of compounds **1b**–**3c** was evaluated by measuring the peak area or retention time of the compound after incubation at varying pH conditions up to 8 h. No degradation was observed at any pH condition for all tested compounds, indicating significant stability. Additionally, all tested compounds were found to be stable for at least 8 h to harsh acid-catalyzed hydrolysis (pH = 2.0, 37 °C, data not shown). Literature studies have shown that the electron-withdrawing nitro group conjugated with the carbamate linker resulted in a remarkable decrease in stability, which may explain the apparent low inhibitory potency of these compounds toward fatty acid amide hydrolase (FAAH), due to decomposition under the assay conditions [33]. Of all compounds (**1b**–**3c**), the nitro group was not in direct conjugation with the carbamate linker, thereby showing no stability loss when incubated under various pH conditions.

### 2.4. Biological Assays

#### 2.4.1. Cell Viability and Clonogenic Assays

**Figure 1:**
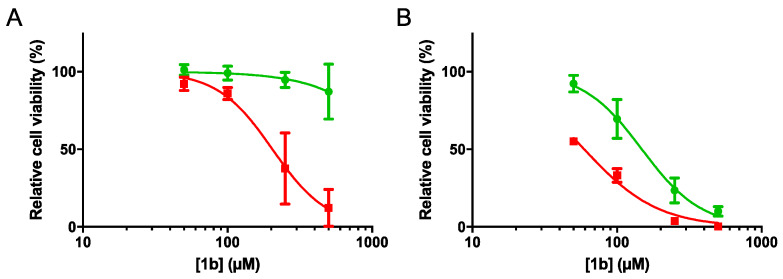
Relative cell viability (%) in (**A**) HT29 and (**B**) HCT116 cells exposed to increasing concentrations of the derivative **1b** under normoxic (green) and anoxic (red) conditions. The data represent the average ± SEM of three independent biological repeats.

The cytotoxicity of all compounds (**1b**–**3c**) was determined in a panel of human tumor cell lines. Compound **1b** showed an IC_50_ of 204.5 μM (*p* < 0.0001) in HT29 cells under anoxia (IC_50_A) and no detectable cytotoxicity (IC_50_ > highest tested concentration) was observed under normoxia (Figure 1). Furthermore, in HCT116 cells, compound **1b** resulted in an IC_50_ of 148.6 and 59.3 μM under normoxic and anoxic conditions, respectively, resulting in a hypoxia selectivity cytotoxicity ratio (HCR) of 2.5 (Table 2). All other compounds, except compound **3b** in HCT116 cells, did not show any cytotoxicity at the tested concentrations. Compound **3b** resulted in a cell-dependent cytotoxicity with an HCR of 2.7 in HCT116 cells, while no cytotoxicity in HT29 cells was observed.

**Figure 2:**
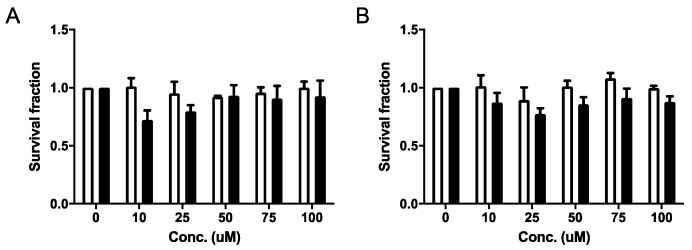
Clonogenic cell survival of (**A**) HT29 and (**B**) HCT116 cells during normoxia (white bars) and anoxia (black bars) when exposed to compound **1b.** The data represent the average ± SEM of three independent biological repeats.

Based on its selective cytotoxicity under anoxia in both cell lines, compound **1b** was selected for further studies, investigating its effects on cell survival. Compound **1b** did not reduce clonogenic cell survival under normoxia or anoxia at the tested concentrations (Figure 2). TH-302, a 2-nitroimidazole based hypoxia-activated prodrug-alkylating agent was used as positive control (data not shown) [34].

#### 2.4.2. Toxicity Evaluation on Zebrafish

During development, zebrafish embryos are easily affected by chemical compounds compared to adult zebrafish or other animal models or cell models and are therefore suitable for assessing the subtle toxic effects of chemicals [28,30]. The toxicity of compound **1b**, **4b** and **5b** was determined by using 24-h post-fertilization zebrafish embryos [35]. In these tests, the LC_50_, zebrafish phenotypic parameters and the swim pattern were analyzed for each compound.

##### Determination of Half Maximal Lethal Concentration 50 (LC_50_)

**Figure 3:**
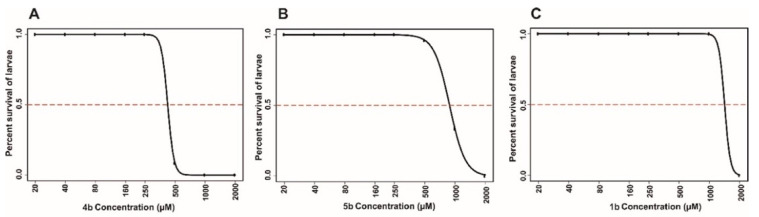
Lethal concentration 50 (LC_50_) values of the prodrugs. The LC_50_ doses of the compounds were calculated based on the 50% mortality of the developing larvae at the end of five days after the exposure of embryos to different concentrations of inhibitors. (**A**) The ranges of LC_50_ values of compound **4b** (<500 μM concentration). (**B**) The LC_50_ value for compound **5b** was below 1000 μM and (**C**) compound **1b** showed an LC_50_ value of about 1400 µM. The LC_50_ doses were determined after three independent experiments with similar experimental conditions (N = 72 larvae).

The lethality of **1b**, **4b** and **5b** on the developing zebrafish embryos was concentration dependent (Figure 3). Among the three compounds tested for the toxicity, **1b** was the less toxic and did not cause any mortality of the larvae, even at a 1 mM concentration at the end of 5 day of exposure to the compound. Compound **4b** was more toxic compared to the other two compounds and caused significant mortality even at a 500-μM concentration (Figure 3). The LC_50_ values of the prodrugs at the end of the 5 days of exposure were in the range of 500 (**4b**), 1000 (**5b**) and 1400 μM (**1b**), as shown in Figure 3. The LC_50_ concentration of the compounds were higher compared to the inhibitors that we screened in our earlier studies [36,37], suggesting that these compounds can be characterized further for developing as drugs.

**Figure 4:**
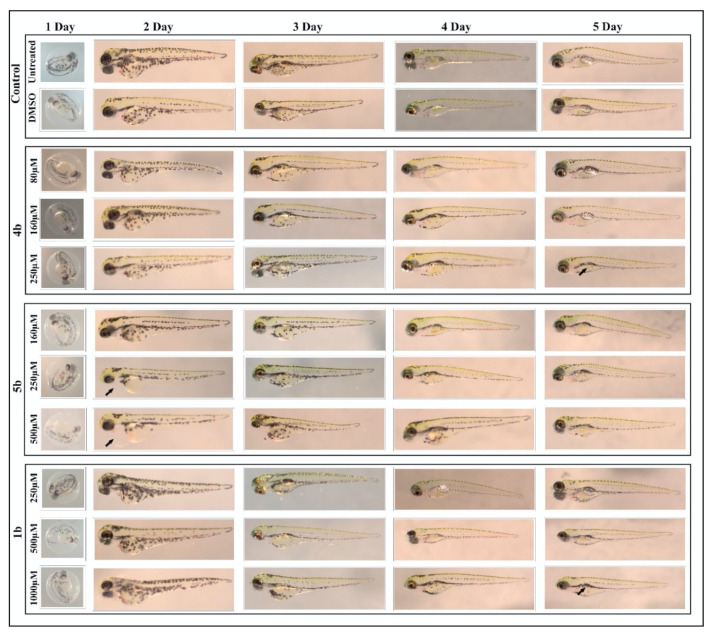
Images of zebrafish larvae in the control and prodrug-treated groups. Representative images of 2–5 dpf zebrafish larvae exposed to different concentrations (80 μM–1000 μM) of **1b**, **4b** and **5b**. The upper panel shows images for normal development of zebrafish larvae in the control group (not treated with inhibitors) and the 1% dimethyl sulfoxide (DMSO)-treated group. The lower panel shows the larvae treated with concentrations of the compounds at which they induced minimal or no phenotypic defects. Prodrug **4b** showed (arrow) an absence of a swim bladder at a 250-μM concentration at 5 days. Prodrug **5b** induced edema (arrow) as early as 2 days post exposure to the compound. Compound **1b** showed an absence of a swim bladder (arrow) at a 1000-μM concentration at 5 days.

Different phenotypic parameters were also analyzed (hatching, heartbeat, edema, swim bladder development, yolk sac utilization and body shape) from the developing larvae of 1–5 days, after exposure to the compounds. Figure 4 shows the representative images of larvae treated with the concentrations considered safe for further characterization. Among the compounds screened, the prodrug **1b** was found to be less toxic with no or minimal phenotypic abnormalities, even at 1 mM. In our earlier study, compounds **1b**–**3c** also showed minimal or no phenotypic abnormalities at 1 mM concentrations. In the present study, compounds **4b** and **5b** showed phenotypic defects, such as edema and the absence of a swim bladder (arrows) at lower concentrations compared to **1b**, as shown in Figure 4.

**Figure 5:**
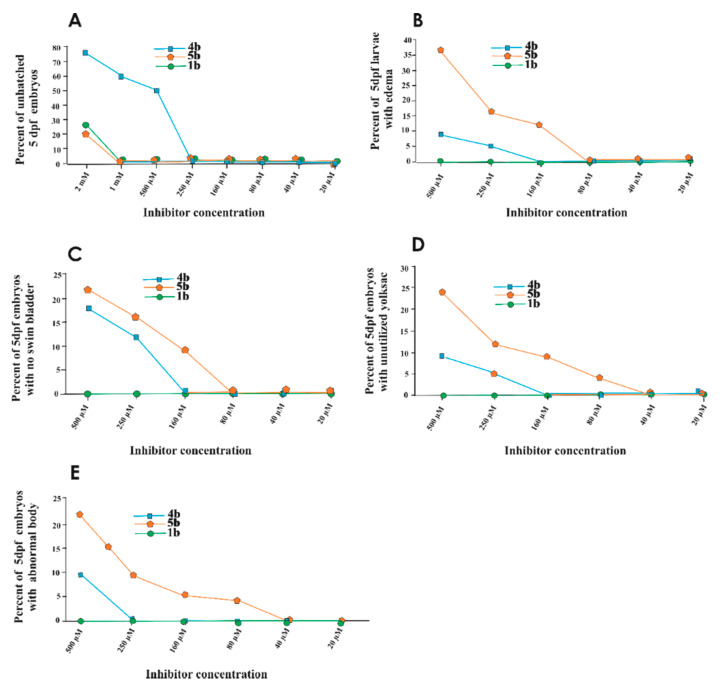
Effect of the prodrugs **4b**, **5b**, and **1b** on the phenotypic parameters of zebrafish larvae. The plot graphs show the phenotypic abnormalities in the zebrafish larvae after 5 days of exposure to compounds. (**A**) Hatching, (**B**) edema, (**C**) swim bladder development, (**D**) yolk sac utilization and (**E**) body shape for each concentration (N = 72).

Furthermore, the toxic effects of compounds across the concentrations (20 μM–2 mM) were assessed on individual parameters of the 5 days post exposed zebrafish larvae. Figure 5 presents plot graphs of the dose-dependent effects of prodrugs on larvae. The results indicate that the **1b** compound showed minimal or no adverse effects on the observable parameters of the larvae (Figure 5A–E). However, prodrugs **4b** and **5b** exhibited adverse effect on hatching, swim bladder development, the utilization of the yolk sac, and the shape of the body and induced pericardial edema, as shown in Figure 5A–E.

##### Swim Pattern Analysis to Assess the Subtle Toxic Effects of the Prodrugs

To assess the subtle toxic effects of the prodrugs on the swim patterns of the larvae, they were assessed at the end of 5 days of exposure to the compounds. The swim pattern analysis showed no abnormal or ataxic movement pattern in the larvae exposed to the concentration (160 µM) that did not induce any of the phenotypic defects and is considered to be safe. In our earlier studies, the nitroimidazole-based compounds DTP338 and DTP348 showed an ataxic movement pattern even at a 100-μM concentration due to neurotoxicity [28,30]. Therefore, the prodrug screening in the current study can be considered safe for further preclinical characterization and development as potential drugs.

## 3. Discussion

We developed here new HAPs incorporating a benzenesulfonamide through a carbamate linker, designed with different bio-reducible moieties for the selective delivery of anticancer drugs on hypoxic tumors. Through this study, we demonstrated that these HAPs can selectively inhibit the hCA IX at the nanomolar level. In contrast to other nitroaromatic drugs, HAPs **1b–3c** did not show stability loss when incubated under various pH conditions as the nitro group was not in direct conjugation with the carbamate linker. Compound **1b**, harboring a 2-nitroimidazole as a reducible moiety, showed the best cytotoxic action against HT29 and HCT116 cells (IC_50_ of 204.5 and 59.36 µM under hypoxia) and an HCR of approximately 2.5. Our data also show that **1b** is the less toxic, with no mortality observed on zebrafish larvae even at a 1-mM concentration at the end of 5 days of exposure. Of note, prodrug **1b** also did not induce phonotypic abnormalities in the larvae as well as abnormal or ataxic swim movement at the concentration that did not induce any of the phenotypic defects and is considered as safe. As other nitroimidazole derivatives, such as DTP338 and DTP348 [30], induced a neurotoxic effect even at a 100-μM concentration, the data gathered here suggest a potential for derivative **1b** to be optimized for the development of new safer HAPs.

## 4. Materials and Methods

### 4.1. Chemistry

General. All reagents and solvents were of commercial quality and used without further purification unless otherwise specified. All reactions were carried out under an inert atmosphere of nitrogen. TLC analyses were performed on silica gel 60 F254 plates (Merck Art. no. 1.05554). Spots were visualized under 254 nm UV illumination or by ninhydrin solution spraying. ^1^H- and ^13^C-NMR spectra were recorded on a Bruker DRX-400 spectrometer (Bruker, Hanau, Germany) using dimethyl sulfoxide-d6 (DMSO-*d*_6_) as solvent and tetramethylsilane as an internal standard. For ^1^H- and ^13^C-NMR spectra, chemical shifts are expressed in δ (ppm) downfield from tetramethylsilane, and coupling constants (*J*) are expressed in hertz. All compounds that were tested in the biological assays were analyzed by high-resolution mass spectra (HRMS) using a Bruker micrOTOF-Q II mass spectrometer (Bruker, Hanau, Germany) fitted with an electrospray ion source

General procedure for preparation of chloroformate: The corresponding hydroxy starting compound (**1a**, **2a** and **3a**) (2 mmol) in tetrahydrofuran (10 mL) was added to phosgene (4 mL, 8 mmol) and tetrahydrofuran (THF) (15 mL) at 0 °C. The reaction mixture was stirred for 16 h, then the solvent was removed in vacuo. The crude chloroformate was used without further purification.

General procedure for synthesis of **1b**, **2b**, **2c**, **3b**, and **3c**: A suspension of chloroformate (23.4 mmol) in dry THF (100 mL) was treated with trimethylamine (50 mmol). The corresponding amine (23.4 mmol) was added afterwards and the reaction was stirred overnight at room temperature. The solvent was removed in vacuo. The residue was dissolved in chloroform and washed twice with 1.0 N sodium hydroxide and water. The organic phase was dried over anhydrous sodium sulfate, filtered and concentrated under vacuum. The residue was purified by chromatography on silica gel using methylene chloride−methanol 98:2 as eluent.

General procedure for synthesis of **4b** and **5b**: To a suspension of carbonyldiimidazole (CDI) (0.31 mmol) in dry THF (5 mL) the corresponding hydroxy starting compound (**4a** and **5a**) (0.28 mmol) was added. The reaction was stirred for 3h at room temperature and the 4-(2-aminoethyl) benzene sulfonamide was added. The reaction mixture was stirred overnight at room temperature and concentrated under reduced pressure. The residue was dissolved in EtOAc (12.5 mL) and washed with water (2 × 7.5 mL) and brine (7.5 mL). The organic phase was dried over anhydrous sodium sulfate, filtered and concentrated under vacuum. The residue was purified by chromatography on silica gel using cyclohexane-ethyl acetate 3:7 as eluent.

*(1-methyl-2-nitro-1H-imidazol-5-yl) methyl (4-sulfamoylphenethyl) carbamate* (**1b**): yield = 92%; ^1^H-NMR (400 MHz, DMSO-*d*_6_) δ 7.73 (d, *J* = 8.3, 2H), 7.48 (t, *J* = 5.6, 1H), 7.38 (d, *J* = 8.3, 2H), 7.30 (s, 2H), 7.21 (s, 1H), 5.12 (s, 2H), 3.89 (s, 3H), 3.26 (dd, *J* = 13.2, 6.7, 2H), 2.79 (t, *J* = 7.1, 2H). ^13^C-NMR (101 MHz, DMSO-*d*_6_) δ 155.36, 145.99, 143.42, 142.09, 133.83, 129.17, 128.41, 125.68, 54.82, 48.62, 34.69, 34.16–33.11. HRMS (ESI+) [M + H]^+^ calculated for [C_14_H_18_N_5_O_6_S]^+^: 384.0978, found: 384.0982.

*2-(2-methyl-5-nitro-1H-imidazol-1-yl) ethyl (4-sulfamoylphenethyl) carbamate* (**2b**): yield = 88%; ^1^H-NMR (400 MHz, DMSO) δ 8.03 (s, 1H), 7.73 (d, *J* = 8.3, 2H), 7.38–7.32 (m, 3H), 7.31 (s, 2H), 4.48 (t, *J* = 4.9, 2H), 4.30 (t, *J* = 4.9, 2H), 3.17 (dd, *J* = 13.1, 6.7, 2H), 2.72 (t, *J* = 7.1, 2H), 2.39 (s, 3H). ^13^C-NMR (101 MHz, DMSO) δ 155.69, 151.86, 143.61, 142.11, 138.56, 133.18, 129.26, 125.81, 61.93, 35.01, 14.02. HRMS (ESI+) [M + H]^+^ calculated for [C_15_H_20_N_5_O_6_S]^+^: 398.1134, found: 398.1136.

*2-(2-methyl-5-nitro-1H-imidazol-1-yl) ethyl (4-sulfamoylbenzyl) carbamate* (**2c**): yield = 56%; ^1^H-NMR (400 MHz, DMSO-*d*_6_) δ 8.04 (s, 1H), 7.88 (t, *J* = 6.1, 1H), 7.76 (d, *J* = 8.4, 2H), 7.34 (d, *J* = 8.4, 2H), 7.32 (s, 2H), 4.54 (t, *J* = 5.0, 2H), 4.36 (t, *J* = 5.0, 2H), 4.19 (d, *J* = 6.1, 2H), 2.44 (s, 3H). ^13^C-NMR (101 MHz, DMSO-*d*_6_) δ 155.87, 151.72, 143.53, 142.68, 138.45, 133.07, 127.26, 125.65, 62.11, 45.52, 43.37, 13.98. HRMS (ESI+) [M + H]^+^ calculated for [C_14_H_18_N_5_O_6_S]^+^: 384.0978, found: 384.0972.

*2-(2-(bis(2-chloroethyl)amino)-5-nitrobenzamido) ethyl (4-sulfamoylphenethyl) carbamate* (**3b**): yield = 40%; ^1^H-NMR (400 MHz, DMSO-*d*_6_) δ 8.72 (t, *J* = 5.3, 1H), 8.15–8.05 (m, 2H), 7.73 (d, *J* = 8.1, 2H), 7.39 (t, *J* = 6.9, 2H), 7.29 (s, 2H), 7.22 (d, *J* = 8.0, 1H), 4.11 (t, *J* = 5.4, 2H), 3.74 (dt, *J* = 9.9, 4.9, 8H), 3.46 (d, *J* = 5.4, 3H), 3.25 (dd, *J* = 13.8, 6.5, 2H), 2.80 (t, *J* = 7.3, 2H). ^13^C-NMR (101 MHz, DMSO-*d*_6_) δ 167.45, 156.11, 151.59, 143.54, 142.05, 138.21, 129.11, 126.33–123.92, 118.08, 62.04, 59.78, 53.12, 41.38, 34.44, 31.23, 30.39. HRMS (ESI+) [M + H]^+^ calculated for [C_22_H_28_Cl_2_N_5_O_7_S]^+^: 575.1008, found: 575.1011.

*2-(2-(bis(2-chloroethyl)amino)-5-nitrobenzamido) ethyl (4-sulfamoylbenzyl)carbamate* (**3c**): yield = 43%; ^1^H-NMR (400 MHz, DMSO) δ 8.75 (t, *J* = 5.5, 1H), 8.17–8.08 (m, 2H), 7.80 (dd, *J* = 12.0, 5.9, 1H), 7.76 (d, *J* = 8.2, 2H), 7.43 (d, *J* = 8.1, 2H), 7.31 (s, 2H), 7.23 (d, *J* = 9.1, 1H), 4.26 (t, *J* = 8.1, 2H), 4.15 (t, *J* = 5.2, 2H), 3.80–3.69 (m, 8H), 3.17 (s, 2H). ^13^C-NMR (101 MHz, DMSO) δ 167.56, 156.53, 151.68, 143.90, 142.68, 138.32, 127.41, 125.76, 117.97, 56.19, 53.22, 48.69, 41.33, 21.08. HRMS (ESI+) [M + H]^+^ calculated for [C_21_H_26_Cl_2_N_5_O_7_S]^+^: 562.0930, found: 562.0935.

*(5-nitrofuran-2-yl)methyl (4-sulfamoylphenethyl) carbamate* (**4b**): yield = 55%; ^1^H-NMR (400 MHz, DMSO-*d*_6_) δ 7.73 (d, *J* = 8.4, 2H), 7.71 (d, *J* = 4.2, 1H), 7.63 (t, *J* = 5.6 Hz, 1H), 7.39 (d, *J* = 8.4 Hz, 2H), 7.37 (s, 2H), 6.84 (d, *J* = 4.2 Hz, 1H), 5.08 (s, 2H), 3.25 (t, *J* = 7.1 Hz, 2H), 2.78 (t, *J* = 7.1 Hz, 2H). ^13^C-NMR (400 MHz, DMSO-*d*_6_) δ 156.45, 155.10, 145.30, 142.26, 130.52, 127.00, 113.99, 113.78, 58.50, 42.62, 36.22. HRMS (ESI+) [M + H]^+^ calculated for [C_14_H_16_N_3_O_7_S]^+^: 370.0709, found: 370.0708.

*(5-nitrothiophen-2-yl)methyl(4-sulfamoylphenethyl)carbamate* (**5b**): yield = 88%; ^1^H-NMR (400 MHz, DMSO-*d*_6_) δ 8.05 (d, *J* = 4.2 Hz, 1H), 7.74 (d, *J* = 8.4 Hz, 2H), 7.63 (t, *J* = 5.6 Hz, 1H), 7.40 (d, *J* = 8.4 Hz, 2H), 7.33 (s, 2H), 7.22 (d, *J* = 4.2 Hz, 1H), 5.23 (s, 2H), 3.24 (t, *J* = 7.1 Hz, 2H), 2.82 (t, *J* = 7.1 Hz, 2H). ^13^C-NMR (400 MHz, DMSO-*d*_6_) δ 156.66, 149.35, 145.27, 142.25, 130.53, 127.52, 126.99, 61.48, 42.59, 36.21. HRMS (ESI+) [M + H]^+^ calculated for [C_14_H_16_N_3_O_6_S_2_]^+^: 386.0481, found: 386.0479.

### 4.2. Carbonic Anhydrase Inhibition Assays

An Sx.18Mv-R Applied Photophysics (Oxford, UK) stopped-flow instrument was used for assaying the CA catalyzed CO_2_ hydration activity. Phenol red (at a concentration of 0.2 mM) was used as an indicator, working at the absorbance maximum of 557 nm, with 20 mM HEPES (pH 7.5) as buffer, and 20 mM Na_2_SO_4_ (for maintaining the constant ionic strength), following the initial rates of the CA-catalyzed CO_2_ hydration reaction for a period of 10–100 s. The CO_2_ concentrations ranged from 1.7–17 mM for the determination of the kinetic parameters and inhibition constants. In particular, CO_2_ was bubbled in distilled deionized water for 30 min so that the water was saturated (the concentration at a specific temperature is known from literature). In addition, a CO_2_ assay kit (from Merck, Darmstadt, Germany) was used to measure the concentration in variously diluted solutions obtained from the saturated one (which was kept at the same temperature and a constant bubbling during the experiments). For each inhibitor, at least six traces of the initial 5%–10% of the reaction were used for determining the initial velocity [32]. The uncatalyzed rates were determined in the same manner and subtracted from the total observed rates. Stock solutions of inhibitor (0.1 mM) were prepared in distilled deionized water and dilutions up to 0.01 nM were done thereafter with distilled deionized water. Inhibitor and enzyme solutions were pre-incubated together for 15 min–2 h (or longer, i.e., 4–6 h) at room temperature (at 4 °C for the incubation periods longer than 15 min) prior to assay, in order to allow for the formation of the EI complex. The inhibition constants were obtained by non-linear least-squares methods using PRISM 3 and the Cheng–Prusoff equation, as reported earlier [38,39], and represent the mean from at least three different determinations. hCA I was purchased by Merck (Darmstadt, Germany) and used without further purification, whereas all the other hCA isoforms were recombinant ones obtained in-house as reported earlier [40].

### 4.3. In Vitro Chemical Stability and Analytical Method

The chemical stability of compounds **1b**, **2b**, **2c**, **3b** and **3c** at various pH (6.2, 6.6, 7.0 and 7.4) was measured on a Waters XEVO QTOF G2 Mass Spectrometer (Waters, Etten-Leur, The Netherlands), with an Acquity H-class solvent manager, Flow-Through Needle (FTN) sample manager and Tunable UV (TUV) detector. The system was equipped with a reversed phase C18 column (Waters, Etten-Leur, The Netherlands, acquity PST 130A, 1.7 µm 2.1 × 50 mm i.d.), with a column temperature of 40 degrees. Mobile phases consisted of 0.1% formic acid in water and 90% acetonitrile. FTN-purge solvent was 5% acetonitrile in water. Mobile phase gradient was maintained starting with 5% acetonitrile to 50% acetonitrile for 15 min at a 220-nm wavelength. Stock solutions of compounds were prepared in DMSO and each sample was incubated at a final concentration of 1–5 μM in pre-thermostated buffered solution. The final DMSO concentration in the samples was kept at 1%. The samples were maintained at 37 °C in a temperature-controlled shaking water bath (60 rpm). At various time points, 100-μL aliquots were removed and injected into the High-Performance Liquid Chromatography (HPLC) system for analysis. Mass was measured in positive sensitivity mode with a mass range between 100 and 1000 Da.

### 4.4. Biological Assays

#### 4.4.1. Cells

Human colorectal HCT116 and HT29 carcinoma cells were cultured in Dulbecco’s Modified Eagle Medium (DMEM, Lonza) supplemented with 10% fetal bovine serum and 1% PenStrep. Cells were exposed to anoxic conditions for 24 h in a hypoxic chamber (MACS VA500 microaerophilic workstation, Don Whitley Scientific, Bingley, UK) with an atmosphere consisting of ≤0.02% O_2_, 5% CO_2_ and residual N_2_. Normoxic cells were grown in regular incubators with 21% O_2_, 5% CO_2_ at 37 °C.

#### 4.4.2. Cell Viability Assays

The cytotoxic efficacy of the bio-reducible derivatives was determined based on cell viability assays using alamarBlue^®^ (Invitrogen). In short, HT29 and HCT116 were seeded in 96-well plates and allowed to attach overnight. On the following day, the plates were exposed to normoxia or anoxia and medium was replaced with pre-incubated normoxic or anoxic DMEM. Compounds were dissolved in DMSO (0.5%, Sigma-Aldrich) and final concentrations were made with pre-incubated anoxic or normoxic DMEM and added to the wells after 24 h of exposure. Cells were exposed to compounds for a total of 2 h, after which the medium was washed off and replaced with fresh medium. Cells were allowed to grow for an additional 72 h under normoxic conditions prior to measurement. Cells were incubated with alamarBlue^®^ for 2 h during normoxic conditions, which corresponds with their metabolic function, a measure for cell viability. Fluorescence was measured using plate reader (FLUOstar Omega, BMG LABTECH, Ortenberg, Germany) using a fluorescence excitation wavelength of 540–580 nm.

#### 4.4.3. Clonogenic Assays

Clonogenic survival HT29 and HCT116 cells were seeded with a high density for CA IX-dependent extracellular acidification [41]. Compound **1b** doses were selected from corresponding IC_50_ (anoxic/normoxic) ranging from 10 to 100 μM for the preliminary experiments. These cells were exposed to 23:30 h normoxic or anoxic conditions and 30 min to drugs after which cells were trypsinized and reseeded in triplicate with known cell numbers. Cells were allowed to grow for 9 (HCT116) and 14 (HT29) days to form colonies that were quantified after staining and fixation with 0.4% methylene blue in 70% ethanol. Surviving fractions were calculated and compared to control survival curves produced in the same experiment without compound treatment.

#### 4.4.4. Statistical Analyses

GraphPad Prism (version 5.03, GraphPad Software, San Diego, CA, USA) was used for all statistical analyses. For the cytotoxic compounds, IC_50_ values were estimated with the curve of the log (inhibitor) vs. normalized response (Variable slope).

### 4.5. Toxicity Assay

#### 4.5.1. Preparation of Inhibitor Samples

The hypoxia-activated prodrugs were screened for their toxicity using 24-h post-fertilization zebrafish embryos as described earlier [35]. The compounds were either dissolved in Embryonic medium (5.0 mM NaCl, 0.17 mM KCl, 0.33 mM CaCl_2_, 0.33 mM MgSO_4_, and 0.1% *w/v* Methylene Blue (Sigma-Aldrich, Darmstadt, Germany) or in dimethyl sulfoxide (DMSO) (Sigma-Aldrich, St. Louis, MO, USA) to prepare 100 mM stock solutions. Before the start of each experiment, the series of dilutions were made from the above stock in the embryonic medium.

#### 4.5.2. Maintenance of the Zebrafish

The wild-type adult zebrafish (AB strains) were maintained at 28.5 °C in an incubator [35]. In each breeding tank, about embryos 3–5 pairs of male and female fish were set up overnight for collecting the embryos. The following morning, 1–2-h post-fertilization (hpf) embryos were collected in a sieve and rinsed with embryonic medium and kept the collected embryos in an incubator at 28.5 °C overnight [35]. The toxicity evaluation studies of the compounds were performed using the fish that were 24-hpf. All the zebrafish experiments were performed at the zebrafish core facility, Tampere University, Finland, and according to the protocol used in our laboratory.

Ethical statement. The research unit at Tampere University has an established zebrafish core facility authorized granted by the National Animal Experiment Board (ESAVI/7975/04.10.05/2016). The experiments using developing zebrafish embryos were performed according to the Provincial Government of Eastern Finland Province Social and Health Department Tampere Regional Service Unit protocol # LSLH-2007–7254/Ym-23.

#### 4.5.3. Determination of half Maximal Lethal Concentration 50 (LC_50_)

The LC_50_ values for all the three prodrugs were determined using 24-hpf embryos using 810 different concentrations for each compound. We used a minimum of 30 zebrafish larvae for each concentration of the compound [28,30,36,42]. The 24-hpf larvae were exposed to different concentrations of the inhibitors that ranged from 20 μM to mM. A dose response curve (DRC) was calculated using the DRM (model fitting function) of the DRC R package [43]. The control group constituted an equal number of larvae not treated with any compound and the larvae that were treated with 1% DMSO. Toxicological evaluation studies were performed in 24-well plates (Corning V R Costar V R cell culture plates). In each well, we placed 1–2 24-hpf embryos in 1 mL of embryonic medium containing a diluted inhibitor. For control groups, 1% DMSO diluted in either embryonic medium or embryonic medium with no chemical compound. A minimum of three sets of experiments were carried out for each prodrug. The mortality of the larvae was checked every 24 h until 5 days after exposure to the prodrugs.

#### 4.5.4. Phenotypic Analysis of the Larvae

After exposure to the prodrugs, we evaluated the effects of these compound on the zebrafish larvae by analyzing the following eight phenotypic parameters: (1) mortality, (2) hatching (3) edema (4) swimming pattern, (5) yolk sack utilization, (6) heartbeat, (7) body shape, and (8) swim bladder development. The images of the developing larvae were taken using a Lumar V1.12 fluorescence microscope (Carl Zeiss MicroImaging GmbH, Göttingen, Germany) attached to a camera with a 1.5 lens (Carl Zeiss MicroImaging GmbH, Göttingen, Germany). The images were analyzed with AxioVision software versions 4.7 and 4.8 as described in our standard protocol for the assessment of the toxicity and safety of chemical compounds.

#### 4.5.5. Swim Pattern Analysis

The swim pattern of the zebrafish larvae was studied after 5 days of exposure to these compounds. For the analyses of swim pattern, about 15–20 zebrafish larvae after 5 days of exposure to the compounds were placed in a 35 mm × 15 mm petri dish containing embryonic medium, and the larvae were allowed to settle in the petri dish for 1 min. The movement of the zebrafish larvae was observed under the microscope for 1 min. A short video of about 1 min was taken for each group of larvae that were treated with a concentration of the compound that did not show any phenotypic abnormalities. The swim patterns were compared with the control group zebrafish larvae that were not treated with any inhibitor.

## Figures and Tables

**Table 1:** Inhibitory activity of compounds **1b**–**5b**, **2c**–**3c**, and the clinically used sulfonamide inhibitor acetazolamide (**AAZ**), against hCA I, hCA II, and hCA IX using a stopped flow CO_2_ hydrase assay.

Compounds	*K_I_* (nM) *			Selectivity Ratio
	**hCA I**	**hCA II**	**hCA IX**	***K_I_* hCA II/*K_I_* hCA IX**
**1b**	166.7	30.6	7.6	1.19
**2b**	3.7	4.3	12.1	0.35
**2c**	2.3	4.0	14.1	0.28
**3b**	83.0	3.1	32.3	0.09
**3c**	2179.9	83.7	88.7	0.94
**4b**	83.8	15.5	5.7	2.72
**5b**	64.7	19.8	15.1	1.31
**AAZ**	250	12.0	25	0.48

* Mean from 3 different assays (errors in the range of ±5–10% of the reported values).

**Table 2:** HCR of all compounds IC_50_N *vs*. IC_50_A.

Compounds	HT29			HCT116		
	IC_50_N	IC_50_A	HCR (IC_50_N/IC_50_A)	IC_50_N	IC_50_A	HCR (IC_50_N/IC_50_A)
**1b**	>500	204.5	>2.44	148.6	59.36	2.50
**2b**	>500	>500	-	>500	>500	-
**2c**	>500	>500	-	>500	>500	-
**3b**	>500	>500	-	267.1	97.27	2.74
**3c**	>500	>500	-	>500	>500	-

Abbreviations: IC_50_: Concentration of an inhibitor where the response is reduced by half; Normoxia (N); Anoxia (A); Hypoxia selectivity cytotoxicity ratio (HCR).
